# Symptom palliation with QUADSHOT regimen for advanced cervical cancer patients in Tikur Anbessa Specialised Hospital: a prospective cohort study

**DOI:** 10.3332/ecancer.2026.2085

**Published:** 2026-03-09

**Authors:** Tsion Zebdiwos Chema, Edom Seife Woldetsadik, Girum Tessema Zingeta, Hawi Furgassa Bedada, Mohammed Ibrahim Adem, Jilcha Diribi Feyisa, Winini Belay, Mushonga Melinda, K S Han Kathy, Rebecca Wong, Munir Awol, Bargude Balta

**Affiliations:** 1 Department of Oncology, School of Medicine, Addis Ababa University, Addis Ababa 1000, Ethiopia; 2 Department of Oncology, Saint Paul Hospital Millennium Medical College, Addis Ababa 1271, Ethiopia; 3 Department of Radiation Oncology, Dartmouth Health, Lebanon, NH 03756, USA; 4 Department of Reproductive Health and Health Service Management, School of Public Health, Addis Ababa University, Addis Ababa 1000, Ethiopia; 5 Kingston Health Sciences Center, Queen’s University, Kingston, ON K7L 2V7, Canada; 6 Department of Radiation Oncology, Princess Margaret Cancer Centre, University of Toronto, Toronto, ON M5G 2M9, Canada; 7 Cancer Center, Hawassa University College of Medicine and Health Sciences, Hawassa, Ethiopia

**Keywords:** cervical cancer, palliation, radiotherapy, Ethiopia

## Abstract

**Background:**

Cervical cancer is the fourth most frequently diagnosed cancer and the fourth leading cause of cancer death in women. Most patients present with advanced stages, and curative treatment is not possible. The purpose of this study is to assess the symptom response and treatment-related toxicities in advanced cervical cancer patients treated with the QUADSHOT (QS) regimen.

**Methods:**

This prospective cohort study enrolled patients with histologically proven advanced cervical cancer candidates for hypo fractionated palliative radiation therapy and not candidates for curative treatment. Patients were treated with a QS radiotherapy regimen (3.7 Gy per fraction twice a day, 6 hours apart on Saturday and Sunday only, every 21 days, to a total dose of 44.4 Gy). Response to treatment was assessed at baseline, at 2, 3 and 6 months after treatment completion.

**Results:**

The mean age of the 53 enrolled patients was 56 years (SD, 10.4). Most patients (43 or 81%) had stage IIIB–IVB illness. Of the 50 patients assessed at 2 months, 43 (86%) had a complete response (CR) from vaginal bleeding, 36 (72%) from vaginal discharge and 25 (50%) from pelvic pain. In the 3rd–5th months, 46 patients were assessed; CR rates were 42 (91%), 37 (80%) and 29 (63%) for vaginal bleeding, vaginal discharge and pelvic pain, respectively. In the sixth month, 19 patients were eligible for assessment, and CR rates were 18 (94%), 17 (89%) and 13 (68%), respectively. The most common acute toxicity was fatigue, followed by grade 1 diarrhea. Four patients had grade three bladder complications.

**Conclusion:**

The QS regimen has rapid symptom relief for patients with advanced cervical cancer with minimal toxicities. The QS regimen is recommended for symptom palliation in settings with restricted resources.

## Introduction

Cervical cancer is the fourth most common cancer in terms of both incidence and mortality in women, with an estimated 660,000 new cases and 350,000 deaths worldwide in 2022 [1]. Cervical cancer is the second most commonly diagnosed cancer in women in Addis Ababa, accounting for 14.7% of the total female cancers and with age-standardised incidence rates of 21.7 per 100,000 females [2, 3].

The majority of patients have advanced stages and comorbidities where curative treatment is no longer practical, and palliative radiotherapy becomes the mainstay of care [4]. The choice of fractionation regimen is crucial for these individuals. Conventional long-course radiation treatments are often not feasible due to patient fragility, limited institutional resources and staff constraints. Conversely, hypo-fractionated regimens have the potential to effectively relieve symptoms with fewer hospital stays, shorter treatment durations and better utilisation of available radiation capabilities [5]. In the palliative context for patients with cervical cancer, several radiation fractionation schemes have been employed to manage symptoms and disease control [6, 7]. The Radiation Therapy Oncology Group (RTOG) 8502 showed a hypo-fractionated regimen known as the ‘Quad-Shot (QS)’ schedule, which consists of 44.4 Gy in 12 fractions. This regimen was shown to be efficacious in treating pelvic cancers and to have tolerable toxicity [7]. This schedule offers substantial logistical advantages in settings with limited resources, such as ours and has been demonstrated to result in good tumour regression and excellent palliation of symptoms [7].

Various hypo-fraction regimens are being used by Ethiopian oncologists to alleviate symptoms. Few studies, however, have looked at how these treatment plans affect symptoms. Thus, the purpose of this study was to assess the clinical outcome of the QS regimen in advanced cervical cancer patients, in order to guide fractionation choices that maintain a balance between institutional capacity and patient benefit.

## Methods

### Study design

An institution-based prospective cohort study was conducted at Tikur Anbessa Specialised Hospital (TASH), Oncology Department, which provides radiotherapy (RT), chemotherapy and palliative treatment. The study period is from 1 February 2022, to 30 November 2022. Patients with biopsy-proven advanced cervical cancer having sufficient baseline clinical evaluation and imaging with advanced-stage disease not amenable to curative treatment were included. Patients with pelvic malignancies other than cervical cancer, those who previously received radiation therapy (RT), and those who did not take at least two-thirds of the recommended dosage of RT were excluded.

Eligible patients received external beam radiotherapy (EBRT) using QS regimen (3.7 Gy per fraction twice a day, 6 hours apart on Saturday and Sunday only, every 21 days, to a total dose of 44.4 Gy). Response to treatment was assessed at baseline, at 2, 3 and 6 months after treatment completion (Table 1). Table 1 shows the treatment schedule of QS radiotherapy regimen.

### Data collection procedure

Patients were evaluated before starting RT by treating the Oncologist with the complete history, physical examination, complete blood count (CBC), liver function tests (LFT) and renal function tests. Staging workups with pelvic magnetic resonance imaging (MRI) or computerised tomography (CT) scans of the abdomen and pelvis and metastatic workups with chest X-rays and abdominal ultrasound were done. Performance status, sociodemographic characteristics and presenting symptoms were documented. Symptom assessment of vaginal discharge, bleeding and pelvic pain was evaluated by four ordinal scales before treatment at each cycle of therapy, then at 2, 3 and 6 months post-RT.

The common terminology criteria for adverse events (CTCAEs) were used to evaluate treatment toxicity. Acute toxicity was assessed during the treatment period up to 2 months of post-treatment, and subacute treatment-related toxicity was assessed from initial treatment within 90 days of post-RT. Data sources included direct patient interviews and secondary data from electronic medical records and patient files. Before data collection, a code number was issued to each patient's chart. Two qualified oncology residents gathered the data. The data collection format was pretested, and the necessary adjustments were performed before the actual study.

### Operational definition

Eastern Cooperative Oncology Group's performance status is a scale used to assess how a patient’s disease is progressing, assess how the disease affects the daily living abilities of the patient and determine appropriate treatment and prognosis [18].

**Advanced cervical cancer:** defined according to the International Federation of Gynecology and Obstetrics stage ≥ IIA2, extends to the pelvic sidewall and/or involves the lower third of the vagina, and/or causes hydronephrosis or a non-functioning kidney and/or involves pelvic and/or para-aortic lymph nodes (stage II). Invades the mucosa of the bladder or rectum or extends beyond the true pelvis (stage IVA) and recurrent or metastasis to distant organs such as the liver, lung, bone and so on [19].

**Treatment volume:** The gross lesion responsible for vaginal bleeding or pelvic pain is identified by a pelvic CT scan or MRI finding as the gross tumour volume (GTV). Then the clinical target volume (CTV) is GTV with the addition of a 2 cm margin. Planning target volume is identified as the CTV with the addition of a 1 cm margin, considering the set-up uncertainty and organ motion.

**QS regimen:** Patients treated with EBRT, three-dimensional conformal RT (3D-CRT) used to deliver a dose of 44.4 in 3.7 Gy per fraction on twice a day, at least 6 hours apart, on Saturday and Sunday every 3 weeks (21 days) for the total fractions of 12 [7].

### RT techniques

We used a linear accelerator machine for treatment. Patients were simulated supine in a CT scan after providing informed consent. All patients were treated with 3D-CRT, and a dose was prescribed to the midplane at the central axis.

**Symptom relief**: for vaginal bleeding was defined as complete disappearance or major reduction of bleeding (decreasing by more than 50% from the previous, i.e., by the number of pads), and complete disappearance or major reduction of malodorous vaginal discharge (decreasing by more than 50% from baseline). No change in amount or response less than 50% is no response [20]. Pelvic pain is assessed according to the four ordinal pain scales, as complete relief from pain is no pain, partial relief from pain is a mild to moderate response, and severe pain is progression pain.

**Acute toxicity:** side effects or adverse effects on gastrointestinal, hematological and skin occurring while on treatment or within 90 days from the initiation of treatment [13]. The severity was scaled according to CTCAE.

**Subacute toxicity:** We defined it as an adverse event that occurred within 90 days of post-RT.

### Data analysis

Data entering, coding and cleaning were performed using Epi-Info version 7.0 and exported to Statistical Package for the Social Sciences version 25 for the analysis. Frequency and cross-tabulation were used to check for missed values and variables. Patients' demographic, clinical and histopathologic characteristics were computed using descriptive statistics such as mean, percentage, frequencies and standard deviation.

## Results

### Sociodemographic characteristics

The research involved 53 patients in total. With a mean age of 56 ± 10.4 years, the participants' ages varied from 34 to 82. Vaginal discharge, irregular vaginal bleeding and pelvic discomfort were the most prevalent clinical symptoms among the patients. These complaints were present in all patients, and 11 patients (20.8%) also reported post-coital bleeding. Of the patients, 19 (35.6%) had co-morbidities. HIV infection was the most prevalent comorbidity, accounting for 11 cases (15.1%), followed by hypertension (7 cases, 13.2%) and diabetes mellitus (4 cases, 7.5%).

### Clinical characteristics

The patient’s performance status was ECOG 1 in 43 (81.1%) of patients and ECOG 2 in 7 (13.2%) of patients. One patient (1.9%) had an elevated creatinine level, this was mild and transient and it did not need discontinuation of RT and six patients (11.3%) had anemia found on CBC. However, there were no abnormal findings on LFT. A punch biopsy was performed on each patient to confirm cervical carcinoma histologically. With 49 (92.5%) cases, squamous cell carcinoma was the most predominant histological subtype. Adenocarcinoma came in second with 3 (5.7%) cases, and one (1.9%) case of adenosquamous carcinoma was found. Of the patients, 43 (81%) had stages IIIB-IVB disease. Stages IIB, IIIA and IVB 6 (11%), 4 (8%) and 1 (2%), respectively, make up the remaining stages.

### Radiation treatment-related characteristics

**Figure 1.**
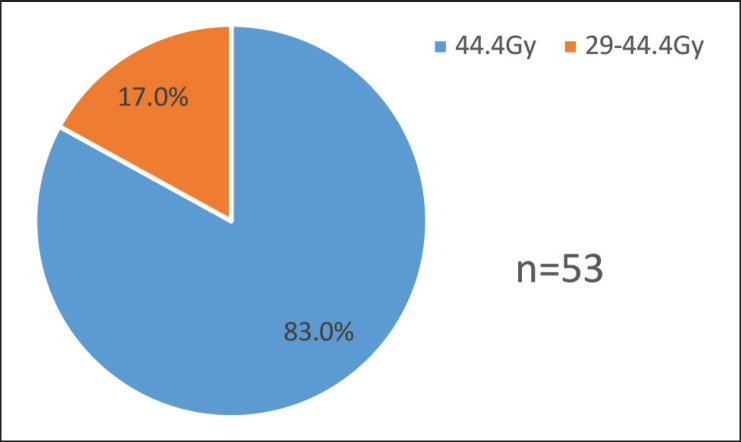
Total doses of radiotherapy delivered for advanced cervical cancer patients treated at Tikur Anbessa Specialised Hospital, 1 February to 31 August 2022, Ethiopia.

For each patient, a QS regimen was administered. Forty-four (83.02%) of the 53 patients received the whole dosage of 44.4 Gy without interruption. The remaining nine (16.9%) patients passed the study's inclusion criteria even though they interrupted treatment after obtaining two-thirds of the recommended dosage because of machine failure, and they received a radiation dose above or equal to 29.4 to 40.4 Gy. The study removed seven individuals who took fewer than two-thirds of the recommended dosages (Figure 1).

Figure 1 shows the total doses of radiotherapy delivered for advanced cervical cancer patients.

### Treatment response evaluation

In terms of symptom relief, all patients had vaginal discharge and bleeding at presentation. Forty-three out of fifty patients (86.0%) had their bleeding completely cease within 2 months after RT. Of the 53, 36 (72.0%) had an improvement in offensive vaginal discharge. Of the 50 patients, about 25 (50.0%) had relief from their pelvic pain.

In 27 (54.0%) of the patients, a pelvic examination revealed no findings. 42 (92.2%) of the patients experienced a complete cessation of vaginal bleeding throughout the next 3 months. 37 individuals or 80.4%, had an improvement in vaginal discharge. Of the patients, 29 showed improvement in their pelvic pain (63.0%). During the last 6 months of follow-up, 17 patients (89.4%) reported no vaginal discharge, and 18 patients (94.7%) reported no vaginal bleeding (Table 2). Table 2 shows the treatment response at 2nd, 3rd and 6th months follow of the patients.

### Treatment-related adverse events

Based on CTCAE, acute complications were detected in 23 (43.4%) of the study participants. The most common acute complication was fatigue, which was seen in 10 (18.9%) patients, followed by Grade 1 diarrhea seen in 9 (17%) patients, and nausea and vomiting seen in 3 (5.7%) patients. After the first cycle and within 6 weeks of RT, 9 (17%) of the individuals had subacute complications. Four patients (7.5%) had Grade 3 bladder problems, which were the most prevalent, followed by two patients (3.8%) who had Grade 2 proctitis. RTOG standards for late radiation morbidity were used to classify subacute toxicity for the bladder, colon and pelvic subcutaneous tissue (Table 3). Table 3 shows treatment-related adverse events seen in cervical cancer patients treated with QS regimen.

## Discussions

Patients with advanced cervical cancer frequently have uncontrolled vaginal bleeding, discharge or pelvic pain; whether or not they are treated by curative intent or not, these individuals require RT to control the symptoms. The best treatment for tumour-related vaginal bleeding, discharge or pelvic discomfort is RT. Palliative treatment should have good symptomatic control, be inexpensive, and have fewer toxicities.

Our study's findings revealed that over 88.6% of patients with RT were in stages III and IV, with 23 (43.3%) and 24 (45.2%) in each category. Advanced disease at presentation has been the major burden in patients with cervical cancer; the current number is higher than the study done at TASH about 57% [24], Ghana (60.4) and Tanzania (65.9%) [21–23]. This is because the results do not truly reflect the stage distribution of cervical cancer in our setting. After all, we selected individuals already in advanced stages of symptom alleviation.

QS RTOG 8502, administered as an outpatient regimen in 12 days spaced 3 weeks apart, decreased the frequency of hospitalisations for treatment and reduced the demand from both patients and carers [10]. In our context, where most of our patients (75.5%) are from rural locations outside of the capital city of Addis Ababa, the shorter treatment duration makes it desirable.

The results of our study showed that in the second month of follow-up, there was symptomatic alleviation of both vaginal discharge (72.0%) and bleeding (86.0%). The complete disappearance of vaginal bleeding (92.2%) in patients and (94.7%) at 3 and 6 months’ follow-up, respectively. Malodorous vaginal discharge completely disappeared in 80.4% of patients at 3 months and 89.4% after 6 months of follow-up. When comparing this study to the groundbreaking RTOG 8502, which also found a 32% overall response (complete response (CR) + partial response), gynecological malignancies accounted for 39.4% of all pelvic malignancies in the study; for patients who finished all three sessions, the response increased to 45%. However, the RTOG 8502 study may have had a lower response because it relied on objective measurements (physical examination and imaging), which were challenging in our case due to a lack of resources for follow-up imaging and the inability to determine physical findings for certain patients [10, 11].

Our study's findings were comparable to those of a study done at M. D. Anderson Hospital on the palliation of advanced gynecologic malignancies (77 out of 111 patients had cervical cancer) using single doses of 10 Gy per fraction spaced 3–4 weeks apart. A significant to total (75%–100%) disappearance of signs and symptoms was observed. Patients receiving two or three single doses had nearly total relief from vaginal bleeding and discharge, and discomfort and edema were rather well-palliated [20].

The second month of follow-up showed a 47.2% improvement in pelvic pain, while the third and sixth months showed 63.0% and 68.4% decrease in pain, respectively. In addition, patients were also receiving analgesics, mostly weak and strong opioids, but the pain-relieving impact was minimal, which may be due to the disease involvement in upper regions like nodal and renal areas, ultimately being outside the irradiation field. Expanding the treatment volume to an area bigger than the existing one should be explored for patients with nodal metastases outside of the pelvis who have severe pain and progression; however, the treating physician should assess the risks and benefits [6, 14]. A loco regional anal cancer subject treated with QS in 2019 who was not a candidate for definitive CRT had symptomatic pain reduction; the regimen is both efficacious and safe for palliation in patients with other pelvic malignancies like anal carcinoma [15].

The pelvic examinations of 27 (54%) of the individuals in our research showed no results, which is a great response. Further studies aiming at this outcome as the primary goal should be considered, since this regimen represents the potential for a clinical (objective) CR. High-dose palliative QS and two high-dose rate conformal brachytherapy treatments produced 82.1% control at the primary site, according to the study's results, which also included two cycles of brachytherapy for patients with stable local disease and good performance status following the QS. Some of the patients in our research were suitable for brachytherapy, but they were not given it due to a lack of machines. In advanced-stage cervical cancer, this regimen provides a durable palliative treatment [14].

Hypo fractionated radiation, as described in the QS regimen (RTOG 8502), not only produces a dosage that effectively relieves symptoms, but it also produces long-term pelvic control. This regimen was well-tolerated and also had the advantage of requiring less time and resources from the patient [10]. Controlling symptoms, especially vaginal bleeding or discharge, is the aim of palliative treatment for cervical cancer. The findings from our study also showed significant symptom relief when compared to the study done by Onsrud *et al* [11]. 10 Grey per fraction was introduced and showed excellent symptomatic control; vaginal bleeding stopped in 90% and malodorous discharge in 39% of the patients.

The 10-Gy single-fraction pelvic radiation regimen is an effective means of symptom palliation and is well tolerated in patients with cancer of the uterine cervix or corpus. However, the interval of symptom control is short, with recurrence within the irradiated field or early progression necessitating re-irradiation. In comparison, the QS regimen takes a more fractionated approach, enabling total doses of up to 44.4 Gy over three cycles, which has been linked to longer-term symptom alleviation and greater local control [2].

Our study's results show similar advantages to other popular palliative RT protocols used in various settings. Using 20 to 25 Gy (median, 25 Gy) in 5 Gy daily fractions, the retrospective study assessed the feasibility and effectiveness of short-course hypofractionated RT for the palliation of uterine cervical cancer. The overall response rates for pelvic pain and vaginal bleeding control were 66.7% and 93.8%, respectively. A short-course regimen of 20 Gy administered in 5 fractions over 1 week is commonly utilised for palliative care of advanced cervical cancer. This regimen gives consistent symptom alleviation with low toxicity, but it frequently requires patients to attend every day for a whole week, which can be difficult for people who travel long distances or have restricted mobility. The QS regimen offers a comparable palliation and provide better logistical convenience when treatment resources and patient transport are restricted [6].

In resource-constrained settings, a systematic review was conducted to determine the best palliative RT dose and fractionation method for treating symptomatic soft tissue pelvic masses. The findings indicated that treatment methods with lower costs and optimal palliation, such as brief hypofractionated ones, are likely to improve access to care [9].

When patients returned for the subsequent cycle, the 3-week break from our present regimen was effective in reducing acute toxicity and relieving symptoms. The effect of the rest time in the QS regimen highlighted questions about tumour repopulation and how it affects normal tissue response. In order to ascertain if the duration of rest would affect tumour response or patient toxicity, RTOG 8502 was randomised between rest intervals of 2 and 4 weeks. There was not a significant difference in tumour response or late complications between the 2- and 4-week rest periods [13]. Acute toxicity of grades 3 or 4 was absent; however, one patient experienced grade 2 diarrhea. The most frequent adverse effect, fatigue (18.9%), can be attributed to the disease's advanced stage. The initial adverse effects had diminished by the time the patient showed up for the subsequent radiation cycle, making this regimen quite practicable. According to a phase III study of accelerated split-course palliative radiation for advanced pelvic malignancies (RTOG-8502), which assessed the effect of rest interval on tumour and normal tissue response, patients with shorter rest intervals tended to experience higher acute toxicity, making the 3-week interval suitable [13].

Only 4.5% of individuals experienced bladder complications of grade 3. The short follow-up period could be the reason for this low number when compared to previous research. In RTOG 8502, the late complication rate peaked at 6.9% at 18 months after starting at 6% at 6 months. Compared to 49% for a similar group of patients receiving a higher dosage per fraction (10 Gy X 3), which was piloted by RTOG-7905, this indicates a considerable reduction in late problems with this regimen. Long-term follow-up of late complications is recommended in our study [7, 8].

The majority of our patients are in the 60–82 age range. In elderly and fragile patients, this regimen has demonstrated manageable side effects and symptom management. In the elderly, hypo fractionated regimens are well tolerated, either with or without brachytherapy, and are a good substitute for radical treatment in individuals with low-risk diseases [26].

Delivering the QS regimen on weekends has both practical benefits and significant problems. Weekend treatments in resource-constrained environments might assist maximise radiotherapy equipment utilisation by lowering daily patient load and avoiding scheduling conflicts with curative cases. This can be especially useful in high-volume environments when machine time is a major bottleneck. However, this technique may necessitate considerable operational changes, such as additional staff, overtime compensation or adjusted work patterns. These characteristics may raise institutional labour costs, requiring significant administrative assistance to ensure sustainability.

From the patient's standpoint, the necessity for two consecutive therapy days every 21 days may provide difficulties, particularly for individuals who reside far from treatment facilities or have financial constraints. Nonetheless, as compared to longer standard schedules, the QS regimen is more practical overall and provides the benefit of controlled, short treatment intervals with the promise of early symptom alleviation.

Weekend delivery was made possible in our context due to institutional flexibility and the commitment of radiation professionals. To ensure the regimen is both clinically successful and operationally sustainable, centers should review their labour capabilities, financial resources and patient logistics prior to widespread implementation.

The study's limitations were that it only covered a small number of patients since we were unable to recruit more because of a machine breakage. Because the variables are subjective, there is a possibility of inter-observer variance and patient over- or under-reporting. The study's strength is that it was carried out at the biggest comprehensive cancer center in Ethiopia and its prospective nature.

## Conclusion

The QS hypo fractionated regimen maintains a balance between treatment efficacy, patient convenience and institutional efficiency, making it an appealing alternative for palliative therapy of advanced cervical cancer in low- and middle-income countries.

Both the incidence of subacute toxicity and the acute toxicities are low, and the treatment is well tolerated. Based on our study's findings, we recommend that the QS regimen be used going forward in the radiotherapy department for the treatment of advanced cervical cancer. Further study is necessary to assess objective tumour response and late toxicity.

## List of abbreviations

CT, Chemoradiation; QS, Quad-shot; RT, Radiation therapy; RTOG, Therapy Oncology Group.

## Conflicts of interest

The authors declare no competing interests.

## Funding

No funding was received for this research.

## Consent for publication

Not applicable.

## Ethical approval

Ethical approval was obtained from the research and ethics committee (REC) of the School of Medicine, College of Health Sciences, Addis Ababa University (Ref No: 001/23/12/2021). Confidentiality and anonymity of the study were maintained. Study participants were involved voluntarily, and written informed consent was obtained from each participant after explaining the purpose of the study.

## Data availability

All data generated or analysed during this study are included in this manuscript and its supplementary information files.

## Author contributions

TC, MI and EW conceptualised the study; HF, TC, MA and JD were involved in the data curation, methodology. KK prepared the figure. TC and MM project administration and analysis. HB and EW were involved in the visualisation, and BB and MA took part in the writing of the original draft WB, MM, RW, TG and BB reviewed and edited the manuscript preparation.

## Figures and Tables

**Table 1.** The treatment schedule of QS radiotherapy regimen.

Cycle	Treatment days	Fractions per day	Dose per fraction (Gy)	Total dose per cycle (Gy)	Interval between cycles	Cumulative dose (Gy)
1	Saturday–Sunday	2 (6 hours apart)	3.7	14.8	21 days	14.8
2	Saturday–Sunday	2 (6 hours apart)	3.7	14.8	21 days	29.6
3	Saturday–Sunday	2 (6 hours apart)	3.7	14.8		44.4

**Table 2.** The treatment response at 2nd, 3rd and 6th month follow of the patients took QS at Tikur Anbessa specialised hospital, February 2022 to 31 August 2022, Ethiopia.

Symptom relief	2nd month follow up, *n* (%)	3rd–5th month follow up *n* (%)	6th month follow up *n* (%)
Bleeding	43 (86.0%)	42 (92.2%)	18 (94.7%)
Discharge	36 (72.0%)	37 (80.4%)	17 (89.4%)
Pain	25 (50.0)	29 (63.0%)	13 (68.4%)
Pelvic finding	27 (54.0%)		
Local progression		12 (26.0%)	
Distant metastasis		6 (13.0%)	4
lung		4	¾
liver			¼
Other		2	
Death Total	3	4	1 8
Not evaluable			26 (not reached 6th month follow-up)
Number of patients	50	46	19

**Table 3.** Treatment related adverse events seen in cervical cancer patients treated with QS regimen in Tikur Anbessa Specialised Hospital, 1 February 2022 to 31 August 2022, Ethiopia.

Variables	Categories	Number (%)
Acute complication (*N* = 23)	Grade 1 Diarrhea	9 (17%)
	Grade 2 Diarrhea	1 (1.9%)
	Nausea and vomiting	3 (5.7%)
	Fatigue	10 (18.9%)
Late complication (*N* = 9)	Grade 1 proctitis	1 (1.9%)
	Grade 2 proctitis	2 (3.8%)
	Grade 1 bladder	1 (1.9%)
	Grade 2 bladder	1 (1.9%)
	Grade 3 bladder	4 (7.5%)
